# Health promoting practices and personal lifestyle behaviors of Brazilian health professionals

**DOI:** 10.1186/s12889-016-3778-2

**Published:** 2016-10-24

**Authors:** Karen D. Hidalgo, Grégore I. Mielke, Diana C. Parra, Felipe Lobelo, Eduardo J. Simões, Grace O. Gomes, Alex A. Florindo, Mário Bracco, Lenildo Moura, Ross C. Brownson, Michael Pratt, Luiz R. Ramos, Pedro C. Hallal

**Affiliations:** 1 JFK Johnson Rehabilitation Institute, JFK Medical Center, Edison, NJ USA; 2 Postgraduate Program in Epidemiology, Federal University of Pelotas, Pelotas, Brazil; 3 Program in Physical Therapy, School of Medicine, Washington University in St. Louis, St. Louis, MO USA; 4 Hubert Department of Global Health, Rollins School of Public Health, Emory University, Atlanta, GA USA; 5 School of Medicine, Department of Health Management and Informatics, University of Missouri, Columbia, MO USA; 6 Gerontology Department, Federal University of Sao Carlos, São Carlos, SP Brazil; 7 School of Arts, Sciences and Humanities, University of Sao Paulo, Sao Paulo, Brazil; 8 Center of Studies and Research Dr. João Amorim, CEJAM, São Paulo, SP Brazil; 9 Pan-American Health Organization, Brasilia, Brazil; 10 Prevention Research Center in St. Louis, Washington University in St. Louis, Brown School, St. Louis, MO USA; 11 School of Medicine, Division of Public Health Sciences and Alvin J. Siteman Cancer Center, Washington University in St. Louis, St. Louis, MO USA; 12 Department of Family Medicine and Public Health, University of California, San Diego, CA USA; 13 Department of Preventive Medicine, Federal University of São Paulo, São Paulo, Brazil

**Keywords:** Health promotion, Lifestyle behavior, Counseling, Health care, Brazil

## Abstract

**Background:**

This study was conducted to examine the lifestyle behaviors and health promoting practices of physicians, nurses, and community health workers in Brazil.

**Methods:**

A random sample of primary health care units in Brazil was selected, and a pretested questionnaire was administered via phone interviews, in 2011, to 182 physicians, 347 nurses, and 269 community health workers, totaling 798 health professionals. The total initial sample included 1600 eligible health professionals. Variables measured included physical activity, alcohol intake, hours of sleep, diet, and perceived self-efficacy to provide preventive counseling on related lifestyle behaviors.

**Results:**

More than 25 % of physicians, nurses, and community health workers reported eating 0–2 portions of fruits and vegetables per day. In terms of cervical and breast cancer, nurses reported to be ‘very prepared’ to advise patients on these topics more frequently than physicians. The prevalence of smoking ranged from 4.9 % among nurses to 7.4 % among community health workers. The proportion of physical inactivity ranged from 40.3 % among nurses to 52.1 % among community health workers.

**Conclusion:**

A reasonably high proportion of physicians, nurses, and community health workers report not engaging in healthy lifestyle behaviors that impact chronic diseases, thus, they may be less likely to encourage such behaviors in their patients.

**Electronic supplementary material:**

The online version of this article (doi:10.1186/s12889-016-3778-2) contains supplementary material, which is available to authorized users.

## Background

Physicians, nurses, and community health workers (CHWs) in Brazil are responsible for managing a plethora of patient health conditions by providing appropriate health promoting recommendations. However, prior research in Latin America and the United States indicates that health professionals are not adequately following health promotion guidelines as it pertains to their own health behaviors [1–3]. It is critical for health professionals to engage in healthy behaviors, including accumulating 150 min per week of moderate to vigorous-intensity physical activity, eating a low risk diet (high in fruits, vegetables, fish, nuts, and seeds), sleeping at least 8 h a day, maintaining low levels of stress, avoiding smoking, and limiting consumption of alcohol, since those behaviors reduce the risk of non-communicable diseases (NCDs) and promote well-being [4]. In addition, it has been shown that when health professionals engage in healthy behaviors they are more likely to provide preventive counseling to their patients [1, 2, 5–7]. Professionals reporting healthy behaviors have higher self-efficacy and confidence when motivating patients to avoid health risk behaviors [1, 2, 5, 7].

Despite documented benefits of the regular practice of physical activity, eating fruits and vegetables, and maintaining a healthy weight, more than 50 % of the adult population of Brazil is overweight, and 60 % do not practice physical activity during leisure time or consume fruits and vegetables regularly [8–11]. In spite of the large amount of information about the effective health benefits of physical activity prescribed by physicians, rates of exercise counseling remain low [1, 11].

A limited amount of research is found in Latin America that examines health professionals’ life style and health behaviors. For instance, a study conducted in Bogota, Colombia, focused on medical students’ health practices but offered no data on the health behaviors of practicing health professionals [12]. However, there is one comprehensive literature review conducted in Africa which focused on the personal physical inactivity and the patient counseling practices among medical doctors [13]. The review targeted individual participation in physical activity and readiness to improve personal health. Another aspect focused on the credibility of the doctors’ counseling and whether they can then disclose personal health practices to patients. It also indicated that physicians’ knowledge on designing an exercise program with the recommended frequencies affected their ability to counsel patients on physical activity [14].

The Brazilian Unified Health System (*Sistema Único de Saúde*; SUS) can potentially take a primary role in implementing evidence based trainings for health care professionals on effective ways to counsel their patients on healthy behaviors and, at the same time, promote uptake of healthy lifestyles among health professionals. Specifically, SUS uses primary health care settings as the first step toward providing prevention and treatment [15]. Additionally, CHWs are an integral part of Brazil’s Unified Health System, meant to provide care funded by taxes to all citizens [16]. The Brazilian goverment also has a strategic plan to tackle the rising rates of NCDs. Thus, it is crucial to place emphasis on the role of health professionals working in primary health care units providing care to around 60 % of the Brazilian population [17].

The purpose of this study is to determine the health promotion practices and personal lifestyle behaviors of health professionals (physicians, nurses, and CHWs) working at primary care units in the Brazilian SUS system. It is hoped that results from this study will increase awareness and help design interventions to improve health professionals’ own lifestyle and health promoting behaviors. The main descriptive research questions include:

1. What percentage of health professionals reported getting at least 8 h of sleep, consuming alcohol within the past month, being physically inactive, and/or watching 4 or more hours of television per day?
2. What percentage of health professionals eat the recommended 5 or more portions of fruits and/or vegetables per day?
3. What percentage of health professionals reported being former or current smokers?
4. How did physicians, nurses, and CHWs perceive their level of preparedness to discuss preventive counseling on five different health topics (nutrition, exercise, weight control, breast cancer, and cervical cancer)?
5. What percentage of nurses vs. physicians recommended that their patients incorporate a healthy diet, practice physical activity, avoid smoking, control alcohol intake, and take medications properly?

## Methods

### Subjects

A list with all 42,000 primary care units in Brazil was compiled. Through a systematic protocol, we randomly selected 1,600 sampling units for inclusion in this study. There were no exclusion criteria as only selected primary care units throughout the 27 states of Brazil were contacted to collect data. In 2011, a health coordinator at each unit and one professional such as a physician, nurse, or CHW was interviewed over the telephone, with random digit sampling, by one of six trained field interviewers. The field interviewers were graduate students from the Federal University of Pelotas in Brazil. The average completion time for the survey was 40 min. The sample included 1,600 eligible health professionals divided as follows: 534 physicians, 533 nurses, and 533 CHWs. The number of health professionals was derived by using the total 1,600 sampling units and dividing it by 3, as our target number of participants in each group of health professionals needed to be distributed as equally as possible. The sample of this study included 182 physicians (34.1 % response rate), 347 nurses (65.1 % response rate), and 269 CHWs (50.5 % response rate), totaling 798 Brazilian health professionals.

### Ethics, consents and permissions

The health professionals were instructed that their participation was voluntary. This cross–sectional study was part of Project GUIA (2010) (Guide for Useful Interventions for Physical Activity in Latin America and Brazil). The Federal University of Pelotas Ethics Committee (#16154) and Washington University in St. Louis Institutional Review Board approved this study.

### Survey questions/items description

The survey had a total of 57 items for CHW respondents and a total of 79 items for physician and nurse respondents (Additional file 1). CHWs were not asked certain items that physicians and nurses had in their questionnaire because it was outside the scope of their job responsibilities. For example, CHWs were not asked if they make the recommendation to their patients with high BMIs or hypertension to control their alcohol intake or items related to medications. Physicians and nurses were asked, “When you treat a patient in a habitual week of work with a certain condition, i.e. high BMI, what do you advise?” The sub-questions included *for the patient to diet*, *to practice physical activity*, *to avoid smoking*, *to control alcohol intake*, and *to take medications properly* (yes vs. no). Physicians and nurses were asked only the questions stated above for the following special patient groups: patients with high BMIs, patients with dyslipidemia altered lipid profile, patients with hypertension, patients with type 2 diabetes, and patients with breast or cervical cancer. Rating-scale items were used to ascertain health professionals’ self-reported personal health behaviors and counseling practices including perceived level of training when talking to patients about healthy lifestyles. Specifically, health professionals’ level of training when talking to patients about nutrition, physical activity, weight control, and breast and cervical cancer screening were rated on a rating-scale from 1 to 3, with 1 representing “unprepared,” 2 representing “somewhat prepared,” and 3 representing “very prepared.” The participants’ body mass index (BMI), which equals weight in kilograms divided by height in meters squared, was calculated using the Centers for Disease Control and Prevention’s standard weight status [18]. Other sociodemographic characteristics included race/ethnicity, marital status, and self-reported health status.

Questions related to lifestyle behaviors, such as alcohol consumption, smoking status, hours of sleep, time spent watching TV, and intake of fruits and vegetables, had discrete response options. For fruit intake, we asked, “On average, how many portions of fruits do you consume per day?” The response format included the following seven options: (0), (1), (2), (3), (4), (5), and (6+). The same was applied to the vegetable intake variable. For time spent watching television, we asked, “How much time do you spend watching TV on a normal weekday (i.e. number of hours)?” The items regarding alcohol use inquired about frequency and daily quantity of alcohol consumption within the past month. One dose or serving was equivalent to 1 can of beer, 1 glass of wine, or 1 shot of hard liquor. According to the USA National Institute on Alcohol Abuse and Alcoholism, men having 5 drinks or more on any single day, and more than 14 drinks per week, is considered heavy or at-risk drinking. At the same time, women having 4 drinks or more on any single day, and more than 7 drinks per week, is considered heavy or at-risk drinking [19, 20].

The survey included physical activity related questions from the International Physical Activity Questionnaire (IPAQ) which has been validated for use in Brazil [21, 22]. All the items were standardized and pilot tested [21, 22]. Specifically, we used the following questions from the IPAQ:

1. “During the last 7 days, on how many days did you do moderate physical activities in your leisure time?” (Additional file 2). Moderate was defined as activities that take modest physical effort and make you breathe somewhat harder than normal.
2. “During the last 7 days, on how many days did you do vigorous physical activities in your leisure time?” (Additional file 2). Vigorous was defined as activities that take hard physical effort and make you breathe much harder than normal. Then, participants were asked from those days how many minutes per day were spent doing moderate-to-vigorous physical activity. Physically inactive participants were those who self-reported not spending at least 150 min per week of moderate-to-vigorous physical activity (leisure time).

### Data analysis

EpiData statistical software version 3.1 [23] was used to enter the collected data, to verify consistency, account for missing values, and then transfer it to Stata version 11 [24]. Descriptive statistics were used to characterize the sample. Age was divided based on size of groups; since only 23 health professionals were 60 years or older, the rest of the age groups were categorized as 20 to 29, 30 to 39, 40 to 49, and 50 to 59. All analyses were stratified by health professional (physician, nurse, or CHW). Multivariate analysis was not performed in this study because the focus of this study was to be descriptive and to highlight possible baseline risk factor behaviors among health care professionals in Brazil. Relative frequencies and percentages of responses across the three groups of health care professionals were calculated. Table 1 includes percentages based on total number of participants for each type of profession, specifically, a physician, nurse, or CHW or overall as appropriate. Table 2 includes a descriptive snapshot of the lifestyle behaviors, such as smoking status, alcohol consumption, duration spent watching television, and personal health behaviors such as consumption of fruits and vegetables, time spent exercising, and sleep duration for the nurses, physicians, and CHWs who responded to the questionnaire.

**Table 1:** Demographic characteristics of health professionals, Brazil, 2011, (*N* = 798)

Variable	Physicians		Nurses		CHWs		Total	
Gender	n	%	n	%	n	%	n	%
Male	103	56.6	53	15.3	29	10.8	185	23.2
Female	79	43.4	294	84.7	240	89.2	613	76.8
*P* value	0.001***				0.698		-	
Age (years)	n	%	n	%	n	%	n	%
20 to 29	44	24.2	164	47.4	76	28.3	284	35.6
30 to 39	59	32.4	117	33.8	107	39.8	283	35.5
40 to 49	32	17.6	44	12.7	58	21.6	134	16.8
50 to 59	31	17.0	18	5.2	24	8.9	73	9.2
> 60	16	8.8	3	0.9	4	1.5	23	2.9
*P* value	0.001***				0.825		-	
Race/Ethnicity	n	%	n	%	n	%	n	%
White	123	67.6	215	62.0	104	39.0	442	55.5
Black	10	5.5	24	6.9	27	10.1	61	7.7
Asian	5	2.8	12	3.5	8	3	25	3.1
Multiracial	44	24.2	96	27.7	127	47.6	267	33.5
Indigenous	0	0	0	0	1	0.4	1	0.1
*P* value	0.636				0.982		-	
Health status	n	%	n	%	n	%	n	%
Excellent	59	32.4	88	25.4	48	17.8	195	24.4
Very good	53	29.1	105	30.3	44	16.4	202	25.3
Good	57	31.3	127	36.6	123	45.7	307	38.5
Regular	13	7.1	22	6.3	54	20.1	89	11.2
Bad	0	0	5	1.4	0	0	5	0.6
*P* value	0.208				0.820		-	
Marital status	n	%	n	%	n	%	n	%
Single	56	30.9	153	44.4	65	24.2	274	34.5
Married/with partner	111	61.3	171	49.6	180	66.9	462	58.1
Separated	12	6.6	19	5.5	17	6.3	48	6.0
Widow(er)	2	1.1	2	0.6	7	2.6	11	1.4
*P* value	0.028*				0.143		-	
BMI	n	%	n	%	n	%	n	%
Below 18.5 (*Underweight*)	6	3.3	16	4.6	10	3.7	32	4
18–24.9 (*Normal*)	75	41.2	196	56.5	128	47.8	399	50.1
25–29.9 (*Overweight*)	72	39.6	91	26.2	92	34.3	255	32
30 and over (*Obese*)	29	15.9	44	12.7	38	14.2	111	13.9
*P* value	0.003**				-		-	

Note: * Statistically significant at *p* < 0.05, ** Statistically significant at *p* < 0.01, ***Statistically significant at *p* < 0.001

**Table 2:** Distribution of Responses for Physicians’, Nurses’, and CHWs’ Lifestyle and Personal Health Behaviors, Brazil, 2011, (*N* = 798)

	Physicians		Nurses		CHWs	
Variable	n	%	n	%	n	%
Sleep duration (hours)						
3–6	89	49.7	94	27.1	61	22.9
7–8	84	46.9	237	68.3	179	67.3
9 (+)	6	3.4	16	4.6	26	9.8
Number of days of alcohol consumption within the last month						
0–3	124	69.3	284	82.1	237	88.4
4–6	40	22.3	47	13.6	22	8.2
7 (+)	15	8.4	15	4.3	9	3.4
Number of doses of alcohol consumption within the last month						
0–2	169	94.9	331	96.2	269	100
3–4	4	2.2	4	1.2	0	0
5 (+)	5	2.8	9	2.6	0	0
Smoked at least 100 cigarettes (lifetime)						
no	143	79.9	305	87.9	216	80.3
yes	36	20.1	42	12.1	53	19.7
Smoking status						
Never	147	82.1	305	87.9	215	79.9
Former	22	12.3	25	7.2	34	12.6
Current	10	5.6	17	4.9	20	7.4
Time spent watching television daily (hours)						
0–1	117	66.1	175	50.6	136	52.3
2–3	52	29.4	150	43.4	101	38.8
4 (+)	8	4.5	21	6.1	23	8.8
Portions of vegetables and fruits consumed						
0–2	50	28	88	25.7	82	30.7
3–4	64	36	156	45.5	115	43.1
5 (+)	64	36	99	28.8	70	26.2
Leisure time/physical activity						
< 150 min	84	46.9	139	40.3	138	52.1

## Results

Table 1 indicates that about 70 % of the participants were in the age range of 20–39 years old and most were females (77 %). However, more male than female physicians responded to the survey (57 %) (Table 1). A large percentage of physicians and nurses identified their race as White, 68 % and 62 %, respectively. As for CHWs, the largest proportion was multiracial (48 %). Most health professionals were married or reported living with a partner (58 %). A large percentage of physicians reported that they perceive their health status to be “excellent” (32 %) whereas (37 %) nurses and (46 %) of CHW rated it as “good.” In terms of BMI, physicians (41 %) and nurses (57 %) were within the normal range (Table 1). Nearly 40 % of physicians were overweight and 16 % were obese (Table 1). As for CHWs, 34 % were overweight and 14 % were obese (Table 1).

**Fig. 1:**
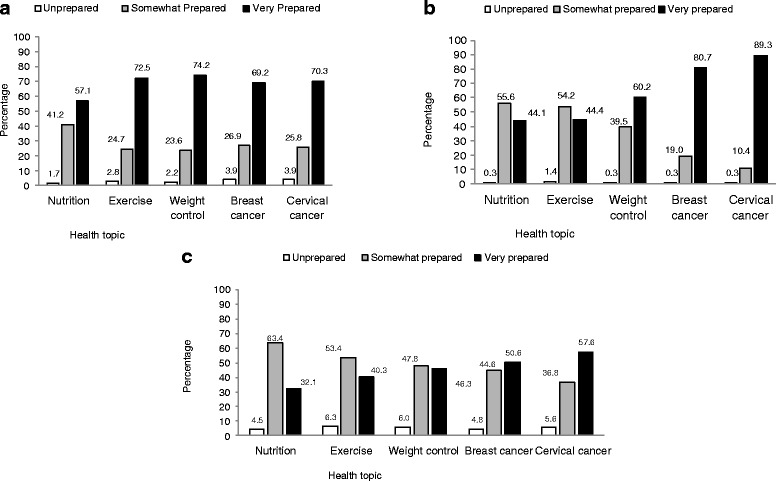
**a**–**c** Health professionals’ perception of knowledge/level of preparedness to discuss preventive counseling on five different health topics with their patients, Brazil, 2011, (*N* = 182 physicians, *N* = 347 nurses, and *N* = 269 CHWs)

Health professionals were asked about their perceived level of training when speaking to patients about nutrition, physical activity, weight control, and breast and cervical cancer screening (Fig. 1a–c). The proportion of CHWs who reported being unprepared or not trained enough to speak with their patients about maintaining a balanced nutrition was 4.5 % (Fig. 1c). The equivalent figures for other topics were: exercise (6.3 %), maintained weight control (6 %), and how to know more about breast cancer (4.8 %) and cervical cancer (5.6 %). Our findings indicate that nurse respondents were more prepared to talk to their patients about breast cancer (80.7 %) and cervical cancer (89.3 %) compared to physicians and CHWs. Compared to nurses and CHWs, a higher percentage of physicians perceived themselves to be prepared to talk to their patients about nutrition (57.1 %), exercise (72.5 %), and weight control (74.2 %). Nearly 41 % of physicians were somewhat prepared to talk to their patients about nutrition, and about 56 % of nurses were somewhat prepared to talk about nutrition. Among the CHWs 63 % responded that they were somewhat prepared to talk to their patients about nutrition. Less than 50 % of CHWs reported they were very prepared to talk to their patients about exercise and weight control, compared to nurses and physicians.

### Lifestyle behaviors

Close to 50 % of physicians reported that they only get 3–6 h of sleep per night. About 68 % of nurses reported receiving about 7–8 h of sleep (Table 2). Compared to nurses (4.6 %) and physicians (3.4 %), a higher percentage of CHWs (9.8 %) reported sleeping 9+ hours. Physicians more often (8.4 %) reported drinking for 7 or more days within the last month compared to nurses (4.3 %) and CHWs (3.4 %). Fewer than 3 % of physicians and nurses reported drinking 5 or more doses of alcohol on one occasion within the last month, and no CHWs reported drinking 3 or more doses of alcohol on one occasion, within the last month.

More CHWs reported being current smokers (7.4 %) compared to nurses (4.9 %) and physicians (5.6 %) (Table 2). Nearly 12 % of physicians and CHWs reported that they were former smokers. Specifically, about 20 % of physicians and CHWs reported having smoked at least 100 cigarettes throughout their lifetime.

In terms of eating vegetables and fruits, about 36 % of physicians reported eating 5 or more portions of fruits and/or vegetables per day, which is the recommended amount by the World Health Organization [25]. About 26 % of CHWs ate 5 or more portions of fruits and/or vegetables. Yet, more than 25 % of physicians, nurses, and CHWs reported eating 0–2 portions of fruits and/or vegetables (Table 2). Furthermore, physicians were the group with the lowest prevalence of watching television for 4 or more hours per day, (4.5 %), followed by nurses (6 %), and CHWs (9 %). During their leisure time, the health professionals reported that less than 150 min per week was spent on moderate-to vigorous physical activity as follows: nurses were 40.3 %, CHWs were 52.1 %, and physicians were 46.9 % (Table 2).

### Health promoting practices/counseling

**Fig. 2:**
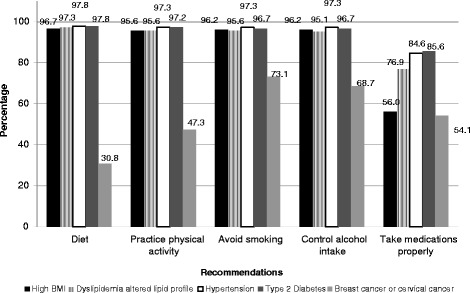
Recommendations physicians give their patients, Brazil, 2011, (*N* = 182)

Some physicians reported not recommending a healthy diet to their patients with breast or cervical cancer (Fig. 2). Specifically, only 31 % of physicians recommended that their patients with breast or cervical cancer maintain a healthy diet. Fewer than 50 % of physicians recommended physical activity to their patients with breast cancer or cervical cancer. Yet, more than 90 % of physicians recommended that their patients with high BMI, dyslipidemia, hypertension, and type 2 diabetes maintain a healthy diet, practice physical activity, avoid smoking, and control their alcohol intake. Fewer than 57 % of physicians who saw patients with a high BMI, breast cancer, or cervical cancer advised them to take medications properly (Fig. 2). About 85 % of physicians advised their patients with hypertension to take medications properly.

**Fig. 3:**
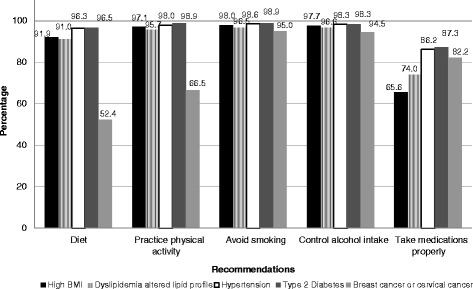
Recommendations nurses give their patients, Brazil, 2011, (*N* = 347)

As for nurses, 91 % recommended that their patients with dyslipidemia maintain a healthy diet. Only 66 % of nurses recommended that their patients with a high BMI take their medications properly. Breast cancer or cervical cancer patients were the groups least likely to receive recommendations about diet from their nurses. Only 52 % of nurses recommended that their patients with breast or cervical cancer maintain a healthy diet (Fig. 3). More than 90 % of nurses recommended that their patients with high BMI, dyslipidemia, hypertension, and type 2 diabetes actively participate in dieting and physical activity, avoid smoking, and control their alcohol intake.

## Discussion

To our knowledge, this study is the first to quantitatively examine the lifestyle and health risk behaviors of physicians, nurses, and community health workers (CHWs) in Brazil. It provides data to measure progress in nationwide initiatives. For example, the Brazilian government embarked on a strategic plan 2011–2022 to address the increasing rates of NCDs. According to the Ministry of Health (2011), the plan’s goal is to decrease the premature mortality rates of NCDs for those under the age of 70 by 2 % per year, to decrease obesity prevalence among children and adolescents, and reduce exposure to risk factors such as smoking, alcohol consumption, lack of physical activity, and salt consumption [26]. “During the development of this national plan, the Brazilian Government took account of the results of evaluation studies of community interventions to promote physical activity, particularly physical activity classes in community settings through programs such as the Academia da Cidade in Recife, Aracaju, and Belo Horizonte” [26] (page 7). In this sense, information from this nationwide survey among health care professionals of Brazil can help to establish a baseline before specific actions and policies are implemented and encouraged by the government. Yet, the descriptive nature of the analysis in this paper, rather than multivariate correlation analyses, which would have allowed the authors to adjust for socio-demographic variables, limits the reach of the conclusions. For instance, it is difficult to discern if many of the differences between nurses, CHWs, and physicians are due to differences in age and gender versus professional backgrounds.

The results from this study will be used as a baseline to understand health behaviors and counseling among primary health care professionals in Brazil and assess the ambitious national plan for reducing population rates of NCDs. A reasonably high percentage of physicians, nurses, and CHWs do not engage in healthy lifestyle behaviors that impact chronic diseases. Based on current evidence, this suggests that they are less likely to encourage these healthy behaviors in their patients [2, 5].

Large differences were found between sleep hours reported by the three groups of professionals. More CHWs got 9(+) hours of sleep (close to 10 %) compared to nurses and physicians (about 5 % and 3 %, respectively); this may be due to a higher prevalence of longer shifts among nurses and doctors. In the United States, sleep deprivation, substance abuse, anxiety, and depression are factors contributing to medical error, as well as to higher rates of work-related injuries for medical residents in training [7, 27]. Thus, it is important to pay attention to the workload and reduced quality and amount of sleep among medical professionals due to the potential harmful impact this can have on the patients. A main finding from this study was that CHWs had the highest prevalence of currently smoking (7.4 %). This may be due to the fact that CHWs do not necessarily have to work at health care institutions in which there are often bans on smoking within or near the facility.

A specific strength of this study is that it reflects the distribution of CHWs in Brazil in relation to age and gender, decreasing the likelihood of selection bias [28, 34]. A CHW can be defined as “any health worker carrying out functions related to health care delivery, trained in some way, in the context of the intervention; and having no formal professional or paraprofessional certificated or degreed tertiary education” [29] (page 3). Because CHWs have less degreed tertiary education compared to nurses and physicians, it is understandable that they may feel unprepared to talk to their patients about how to maintain healthy lifestyles, including nutrition, physical activity, weight control, and counseling on breast and cervical cancers. Breast and cervical cancer are the two most frequent cancer types among women in Brazil [30, 31], which justifies their inclusion in this study. A recent paper on cervical cancer prevention published with data from this survey found that although most primary health care units conducted screening, they also used home visits to conduct recruitment and outreach and provided follow-up. More training and information on effective prevention and screening strategies is needed, particularly for CHWs who offer more direct contact with women at the community level [31].

Certification and training programs for CHW in Brazil that focus on nutrition and physical activity could be implemented to take full advantage of the universal nature of the health care system and their engagement with community members. Moreover, physicians, nurses, and CHWs would greatly benefit from an exchange of information and knowledge regarding real needs and health concerns of the community. However, a review of 109 articles shows that CHWs and medical providers are rarely trained together, limiting opportunities for exchange of knowledge and skills from diverse sources and perspectives [32]. In the United States, continuing education workshops help keep health professionals updated on new public health research and ways to better serve patients and increasingly inter-professional learning (IPL) programs are being encouraged at the institutional and academic level so that healthcare students are better prepared to become cross disciplinary professionals [33]. The results of this study suggest that continuing education workshops and IPL may help increase the percentage of physicians, nurses, and CHWs in Brazil who feel prepared to counsel their patients [34]. Furthermore, another published article that was completed using the data collected from this same study’s questionnaire, found that nearly 40 % of the health professionals in Brazil incorrectly believed ninety minutes of moderate-intensity physical activity per week is the recommended amount for health benefits, when the actual recommended amount by WHO is accumulating 150 min per week of moderate to vigorous-intensity physical activity [35]. This could be part of the government strategy to reduce the incidence of NCD at the national level.

Although cancer was not the main focus of this paper, physicians in this study may not be advising their advanced stage cancer patients to practice physical activity due to a perception that these patients may not have the strength to practice physical activity. Yet, depending on the type of cancer and the stage, physical activity can help a cancer patient. According to the American Cancer Society, research has shown that exercise is not only safe and possible during cancer treatment, but it can improve physical function and quality of life [36–38]. The only time cancer patients should not practice physical activity is when it causes pain, rapid heart rate, or shortness of breath [39].

This study was based on self-report and self-perception data, thus information about socially undesirable behaviors such as smoking and alcohol use may be underreported, limiting the results from this study. The field interviewers interviewed 1,200 respondents, but 400 participants did not respond to all of the questions. A non-monetary reward, such as a fruit basket, for the participants should have been provided for taking the time to answer all the questions, since it could have helped increase the response rate. The distribution of the response rate is not the same within the three health professional groups, which could have also led to response bias, when analyzing the sample in its entirety. The low response rate in this study can be explained by the refusal of health care workers to take time away from their responsibilities and also to answer personal questions about their own lifestyles. Additionally, the survey took approximately 45 min to answer, which is a considerable amount of time for a health practitioner. It is common to have low response rates among health professionals, especially physicians.

It is difficult to determine to what extent health professionals would be willing to admit they are not prepared to counsel their patients on certain topics since most health professionals have an ethical responsibility to stay up-to-date and knowledgeable about best evidence-based practices that can assist their patients. Yet, people are more likely to be honest and open to disclosure when there is greater “social distance,” specifically between respondents and their interviewers [40]. A more distant telephone interviewer will have less of a social influence and will less likely elicit either favorable or unfavorable reactions from the respondent. Thus, one advantage of this study is the use of the telephone based survey since more social desirability bias may occur in face-to-face interviews than over the telephone [40].

In addition, alcohol consumption questions were not specific in the survey, for example, there was no operationalized definition of binge drinking (frequency for which an individual consumes more than three alcoholic beverages in a 4-h period during an average week and dosage effects) [1, 41, 42]. This could have limited our ability to interpret and report the results.

One of the major contributions from this study is that our findings are new and provide current and unique information about health professionals’ behaviors in Brazil. There are many studies about medical and nurse student behaviors, but there are few on practicing physicians, nurses, or CHWs. This study also brings to light base line information in regard to health professionals’ need to improve their lifestyle behaviors so that they can potentially feel more prepared and equipped to counsel their patients. Additionally, our study’s findings can encourage more partnerships and advocacy for changes or improvements in the existing national health policies in Brazil. Other strengths of this study include the selection of a representative group of health care professionals from all 5 regions of Brazil (North, South, North East, South East, and Central West) and a high response rate (more than 50 %) for nurse and CHW participants.

## Conclusion

During the last 10 years, actions have been taken toward developing and implementing more evidence-based approaches for promoting physical activity and chronic disease prevention in Brazil [43]. However, there is scant information about evidence-based approaches targeting health professionals, such as physicians, nurses, and CHWs, advising them to engage in health promoting behaviors in Brazil. Health professionals need to understand why individuals make certain decisions about health risk behaviors. Furthermore, physicians, nurses, and CHWs are encouraged to be more proactive in terms of their own risk behaviors. Health professionals’ attitudes toward physical activity, smoking, and alcohol abuse counseling may be enhanced through exposure to health promotion practices at the workplace and peer social support as well as by formal training [34]. The highlights of this study include that a high portion of health professionals in Brazil do not engage in healthy lifestyle behaviors and our findings may be used to advocate for improvements in existing national health policies in Brazil. A low percentage of health professionals feel prepared or trained to counsel patients about healthy behaviors, a finding that warrants further investigation and educational interventions.

## Additional files

Additional file 1: Project GUIA’s physicians and nurses survey and community health workers survey. (ZIP 3240 kb)
Additional file 2: International physical activity questionnaire. (PDF 29 kb)

## Abbreviations

BMI: Body mass index
CHWs: Community health workers
GUIA: Guide for useful interventions for physical activity
IPAQ: International physical activity questionnaire
IPL: Inter-professional learning
NCDs: Non-communicable diseases
SUS: Sistema Único de Saúde
