# Supplementary material for: Health promoting practices and personal lifestyle behaviors of Brazilian health professionals
Source: BMC Public Health. 2016 Oct 24;16:1114. doi: 10.1186/s12889-016-3778-2 (PMC5078940; doi:10.1186/s12889-016-3778-2)
Supplement: Additional file 1: — Project GUIA’s physicians and nurses survey and community health workers survey. (ZIP 3240 kb) [file 12889_2016_3778_MOESM1_ESM.zip › Project GUIA Survey CHWR1.pdf]

PROJETO GUIA – Inquérito Telefônico nas Unidades Básicas de Saúde

Questionário para agentes comunitários de saúde

BOM (*DIA, TARDE, NOITE*)***. MEU NOME É ...., ESTOU LIGANDO DO CENTRO DE EPIDEMIOLOGIA DE PELOTAS-RIO GRANDE DO SUL. ESTAMOS REALIZANDO UMA PESQUISA COORDENADA PELO MINISTÉRIO DA SAÚDE E ALGUMAS UNIVERSIDADES BRASILEIRAS E DOS ESTADOS UNIDOS.***

Existem agentes de saúde trabalhando nessa unidade? (0) Não → *ENCERRE A ENTREVISTA*

(1) Sim

Você poderia me informar o primeiro nome de cada um deles?

COM BASE NOS NOMES INFORMADOS PELO RESPONDENTE, O ENTREVISTADO DEVE "SORTEAR" SEMPRE O NOME DO MEIO. CASO O NÚMERO DE AGENTES SEJA PAR, SORTEAR O NOME DO MEIO + 1.

GOSTARIA DE FALAR COM O(A) *(NOME DO AGENTE)* ____.

Código da UBS: __ __ __ __

Cidade:____

Estado: __ __

Telefone:_(____)________________________________

Profissional entrevistado: (3) Agente comunitário de saúde

Data da entrevista: ___/___/______

Entrevistador: ______________________________ (código do entrevistador: __ __)

BLOCO DE FORMAÇÃO INICIAL E ATUAÇÃO PROFISSIONAL

1) Você estudou até:

(0) Ensino Fundamental (1º grau) incompleto – *PULE PARA QUESTÃO 4*

(1) Ensino Fundamental (1º grau) completo – *PULE PARA QUESTÃO 4*

(2) Ensino Médio (2º grau) incompleto – *PULE PARA QUESTÃO 4*

(3) Ensino Médio (2º grau) completo – *PULE PARA QUESTÃO 4*

(4) Superior incompleto

(5) Superior completo

(6) Pós-graduação

2) Se você ESTÁ CURSANDO o 3° GRAU ou já COMPLETOU, qual o curso de graduação?

(0) Medicina (1) Enfermagem (2) Serviço Social (3) Nutrição

(4) Odontologia (5) Fisioterapia (8) NSA ( ) Outro ____________

Há quanto tempo você concluiu o 3° grau: __ __ (anos) (88) NSA

3) Você tem:

**Residência médica?**	(0) Não	(1) Sim	(8) NSA
**Residência em enfermagem?**	(0) Não	(1) Sim	(8) NSA
**Especialização?**	(0) Não	(1) Sim	(8) NSA

SE SIM, QUAL ÁREA:____

Mestrado? (0) Não (1) Sim (8) NSA

SE SIM, QUAL ÁREA:____

Doutorado ? (0) Não (1) Sim (8) NSA

SE SIM, QUAL ÁREA:____

4) Qual ano você concluiu a formação de mais alto grau? __ __ __ __

5) Há quanto tempo você trabalha nesta unidade de saúde? __ __ anos __ __ meses

BLOCO DE VARIÁVEIS DEMOGRÁFICAS

6) Qual a sua idade? __ __ anos

7) Como você se classifica com relação à sua cor de pele?

(1) Branco (2) Preto (3) Amarelo (4) Pardo (5) Indígena (6) Sem declaração (9) IGN

8) Sexo do entrevistado (1) Masculino (2) Feminino

9) Qual é a sua situação conjugal atual?

(1) Solteiro (2) Casado/Mora com companheiro (3) Separado (4) Viúvo

VARIÁVEIS RELACIONADAS AO ESTADO DE SAÚDE

10) Qual a sua altura atual (cm)? __ __ __ (999) IGN

11) Qual o seu peso atual (kg)? __ __ __ (999) IGN

SOMENTE PARA MULHERES: **Caso a Sra. esteja grávida, indique seu peso antes de engravidar __ __ __** kg

12) Em geral, como você considera sua saúde?

(1) Excelente (2) Muito boa (3) Boa (4) Regular (5) Ruim

13) Em uma semana normal, quantas pessoas você costuma visitar nas casas? __ __ __ pessoas

14) O quanto você se considera preparado para falar com as pessoas que você atende sobre os seguintes assuntos?

(a) Nutrição/alimentação (1) Sem preparo (2) Um pouco de preparo (3) Bastante preparado

(b) Exercício/atividade física (1) Sem preparo (2) Um pouco de preparo (3) Bastante preparado

(c) Controle de peso (1) Sem preparo (2) Um pouco de preparo (3) Bastante preparado

(d) Câncer de mama (1) Sem preparo (2) Um pouco de preparo (3) Bastante preparado

(e) Câncer de colo do útero (1) Sem preparo (2) Um pouco de preparo (3) Bastante preparado

BLOCO DE BARREIRAS PARA ACONSELHAMENTO EM ATIVIDADE FISICA

Gostaria que você me dissesse se concorda totalmente, concorda, é indeciso(a), discorda ou discorda totalmente com a seguinte frase.

15) Programas voltados à atividade física para a comunidade devem ser oferecidos pelo Sistema Único de Saúde

(1) Discordo Inteiramente (2) Discordo (3) Indeciso (4) Concordo (5)Concordo inteiramente

16) Você considera viável o oferecimento de programas de atividade física na sua unidade de saúde?

(0) Não (1) Sim

17) Você considera prioritário o oferecimento de programas de atividade física em sua unidade de saúde?

(0) Não (1) Sim

18) Em sua opinião, qual o profissional da saúde é o principal responsável pela promoção da atividade física?

(1) Médico (2) Profissional de Educação Física (3) Nutricionista (4) Fisioterapeuta

(5) Enfermeiro ( ) Outro ____

19) Quais destas são dificuldades para você aconselhar sobre a prática de atividade física aos pacientes? Para cada pergunta, por favor, responda sim ou não.

**Falta de conhecimento sobre o assunto**	(0) Não	(1) Sim
**Falta de tempo no atendimento**	(0) Não	(1) Sim
**Falta de locais adequados para a prática**	(0) Não	(1) Sim

Outro:____

20) Indique uma das alternativas abaixo que melhor represente a sua atitude em relação à orientação de atividade física para a saúde das pessoas que você atende:

(1) Eu não recomendo atividade física e não tenho intenção de começar a recomendar

(2) Eu não recomendo atividade física, mas estou pensando em começar a recomendar

(3) Eu recomendo a atividade física algumas vezes, mas não regularmente

(4) Eu recomendo a atividade física regularmente, mas iniciei recentemente

(5) Eu recomendo a atividade física regularmente há mais de 6 meses

(6) Eu recomendava a atividade física no passado, mas agora não

BLOCO SOBRE HÁBITOS RELACIONADOS À SAUDE

NESSA SEÇÃO FAREMOS PERGUNTAS RELACIONADAS AOS SEUS HÁBITOS DE SAUDE

21) Normalmente, quantas horas por noite você dorme? __ __ horas

22) Durante o ultimo mês, aproximadamente, quantos dias você ingeriu bebida alcoólica?

(0) Nenhum – *PULE PARA A QUESTÃO 25* ___ dias

23) Nesses dias, quantas doses você tomou na ocasião? (1 dose equivale a uma lata de cerveja, 1 taça de vinho, 1 coquetel ou 1 copo de licor)

__ __ doses (88) NSA

24) Nesse último mês, aproximadamente, quantos dias você tomou 5 ou mais doses na mesma ocasião?

__ __ dias (88) NSA

25) Você já fumou pelo menos 100 cigarros na sua vida? (5 maços equivalem a 100 cigarros)

(0) Não (1) Sim

26) Em relação ao cigarro, atualmente, você?

(0) Não fuma (1) Fuma todos os dias (2) Fuma alguns dias (3) É ex-fumante.

AGORA NÓS VAMOS FALAR SOBRE ATIVIDADES FÍSICAS/EXERCÍCIOS QUE VOCÊ REALIZA NO SEU TEMPO LIVRE. POR FAVOR, NÃO RESPONDA AQUI SOBRE AS SUAS ATIVIDADES NO TRABALHO.

GOSTARIA QUE VOCÊ PENSASSE EM TODAS AS <u>ATIVIDADES FÍSICAS DE INTENSIDADE FORTE</u> QUE VOCE REALIZOU DESDE *<DIA DA SEMANA PASSADA>* ***NO SEU TEMPO LIVRE****.* ***ATIVIDADES DE INTENSIDADE FORTE SÃO AQUELAS QUE EXIGEM UM ESFORÇO FÍSICO FORTE, FAZEM VOCE RESPIRAR MAIS RÁPIDO QUE O NORMAL E O CORAÇÃO BATER MAIS FORTE QUE O NORMAL. ESSAS ATIVIDADES PODEM INCLUIR: CORRIDA, JOGAR FUTEBOL, PEDALAR RÁPIDO, ENTRE OUTRAS. <u>PENSE APENAS NAS ATIVIDADES QUE TIVERAM DURAÇÃO DE PELO MENOS 10 MINUTOS SEGUIDOS.</u>***

27) Desde <*7 dias atrás***>, quantos dias você realizou atividades físicas de intensidade forte no seu tempo livre?**

(0) Nenhum – *PULE PARA QUESTÃO 29* __ dias da semana

28) Nos dias que você realizou essas atividades, aproximadamente, quanto tempo elas duraram por dia?

__ __ __ minutos (888) NSA

AGORA PENSE NAS ATIVIDADES FÍSICAS DE INTENSIDADE MODERADA QUE VOCÊ REALIZOU NO SEU TEMPO LIVRE. ATIVIDADES FÍSICAS MODERADAS SÃO AQUELAS QUE EXIGEM UM ESFORÇO FÍSICO MODERADO E FAZEM VOCE RESPIRAR UM POUCO MAIS RÁPIDO QUE O NORMAL. ESTAS PODEM INCLUIR: DANÇAR, PEDALAR DEVAGAR, CAMINHAR, ENTRE OUTRAS. PENSE APENAS NAS ATIVIDADES QUE TIVERAM DURAÇÃO DE PELO MENOS 10 MINUTOS SEGUIDOS.

29) Desde <*7 dias atrás*>, quantos dias você realizou atividades físicas moderadas no seu tempo livre?

(0) Nenhum – *PULE PARA QUESTÃO 31* __ dias da semana

30) Nesses dias, aproximadamente, quanto tempo por dia duraram essas atividades?

__ __ __ minutos (888) NSA

31) Desde < *7 dias atrás*>**, quantos dias você realizou caminhadas no seu tempo livre?**

(0) Nenhum – *PULE PARA QUESTÃO 33* __ dias da semana

32) Nesses dias, aproximadamente, quanto tempo duraram essas caminhadas no seu tempo livre?

__ __ __ minutos (888) NSA

AGORA EU GOSTARIA QUE VOCÊ PENSASSE COMO VOCÊ SE DESLOCA DE UM LUGAR AO OUTRO. PODE SER A IDA E VINDA DO TRABALHO OU QUANDO VOCÊ VAI FAZER COMPRAS, VISITAR AMIGOS OU IR À ESCOLA. LEMBRE DE FALAR APENAS SOBRE AS ATIVIDADES QUE DURAM PELO MENOS 10 MINUTOS SEGUIDOS. NÃO CONSIDERE AQUI AS CAMINHADAS QUE VOCÊ FAZ DURANTE A SUA JORNADA DE TRABALHO.

33) Desde < *7 dias atrás*>**, quantos dias você utilizou a bicicleta para ir de um lugar para outro?**

(0) Nenhum – *PULE PARA QUESTÃO 35* __ dias da semana

34) Nesses dias, aproximadamente, quanto tempo duraram essas pedaladas?

__ __ __ minutos (888) NSA

35) Desde < *7 dias atrás*>, **quantos dias você caminhou para ir de um lugar a outro?**

(0) Nenhum – *PULE PARA QUESTÃO 37* __ dias da semana

36) Nesses dias, aproximadamente, quanto tempo duraram esses deslocamentos?

__ __ __ minutos (888) NSA

AGORA VAMOS FALAR SOBRE ASSISTIR TV E ALIMENTAÇÃO

37) Quanto tempo você passa por dia assistindo TV num dia de semana normal?

__ __ horas __ __ minutos

38) Em média, quantas porções de fruta você consome por dia?

(0) (1) (2) (3) (4) (5) (6+)

39) Em média, quantas porções de verduras ou vegetais você consome por dia?

(0) (1) (2) (3) (4) (5) (6+)

PARA AS QUESTÕES SEGUINTES INDIQUE A RESPOSTA QUE VOCÊ JULGAR CORRETA. CASO VOCÊ NÃO ESTIVER SEGURO DA RESPOSTA, INQUIQUE A OPÇÃO "NÃO SEI".

40) O consumo de frutas e verduras que se deve recomendar a um adulto, por dia, é:

(1) Mínino 1 porção de fruta e/ou verdura

(2) Mínino 2 porções de frutas e/ou verduras

(3) Mínino 3 porções de frutas e/ou verduras

(4) Mínino 4 porções de frutas e/ou verduras

(5) Mínino 5 porções de frutas e/ou verduras

(6) Mínino 6 porções frutas e/ou verduras

(7) Não sei

41) O perímetro abdominal (cintura) recomendado para a América Latina para manter um risco baixo de enfermidade cardiovascular e diabetes tipo 2 é?

(1) < 120 cm para homens e de < 110 cm para mulheres

(2) < 110 cm para homens e de < 100 cm para mulheres

(3) < 100 cm para homens e < de 90 cm para mulheres

(4) < 90 cm para homens e < 80 cm para mulheres

(5) < 85 cm para homens e < 75 cm para mulheres

(6) Não sei

42) Como se define sobrepeso e obesidade em adultos?

(1) Um IMC > 20 (sobrepeso) e > 25 (obesidade)

(2) Um IMC > 25 (sobrepeso) e > 35 (obesidade)

(3) Um IMC > 18.5 (sobrepeso) e > 25 (obesidade)

(4) Um IMC > 25 (sobrepeso) e > 30 (obesidade)

(5) Não sei

SOBRE AS RECOMENDAÇÕES ATUAIS DE ATIVIDADE FÍSICA PARA SAUDE

43) Como você considera o seu conhecimento sobre as recomendações atuais de atividade física para a saúde?

(1) Sei o suficiente **(2) Gostaria de aprender mais** **(3) Insuficiente**

44) Quantos dias da semana, no mínimo, as pessoas devem fazer atividades físicas de intensidade moderada para obter benefícios a saúde?

__ dias da semana (88) Não importa (99) Não sei

45) Nos dias em que a pessoa faz atividade física moderada, qual o tempo mínimo recomendado para obter benefícios à saúde?

__ __ __ minutos (88) Não importa o tempo (99) Não sei

46) Para que uma atividade física moderada tenha efeito positivo sobre a saúde ela deve ser feita de que forma?

(1) Deve ser feita em uma única vez durante o dia

(2) Pode ser feita em uma única vez durante o dia ou dividida em 2-3 vezes, de 10 a 15 minutos, durante o dia

(3) Não sei

47) Quantos dias da semana, no mínimo, as pessoas devem fazer atividades físicas de intensidade forte, para obter benefícios a saúde?

__ dias da semana (88) Não importa (99) Não sei

48) Nos dias em que a pessoa faz atividade física forte, qual o tempo mínimo recomendado para obter benefícios à saúde?

__ __ __ minutos (88) Não importa o tempo (99) Não sei

49) Para que uma atividade física forte tenha efeito positivo sobre a saúde ela deve ser feita de que forma?

(1) Deve ser feita em uma única vez durante o dia

(2) Pode ser feita em uma única vez durante o dia ou dividida em 2-3 vezes, de 10 a 15 minutos, durante o dia

(3) Não sei

Gostaria que você me dissesse se concorda totalmente, concorda, é indeciso(a), discorda ou discorda totalmente com a seguinte frase.

50) As pessoas podem combinar atividades moderadas (exemplo: caminhada) com atividades vigorosas (exemplo: corrida) com o objetivo de atingir a quantidade mínima de atividade física para a saúde.

(1) Discordo Inteiramente (2) Discordo (3) Indeciso (4) Concordo (5)Concordo inteiramente

51) Você acha que a falta de atividade física, sedentarismo, pode causar:

**Diabetes tipo 2?**	(0) Não	(1) Sim	(9) Não sabe
**Pressão alta?**	(0) Não	(1) Sim	(9) Não sabe
**AIDS?**	(0) Não	(1) Sim	(9) Não sabe
**Osteoporose, fraqueza nos ossos?**	(0) Não	(1) Sim	(9) Não sabe
**Câncer de pulmão?**	(0) Não	(1) Sim	(9) Não sabe
**Depressão?**	(0) Não	(1) Sim	(9) Não sabe
**Cirrose?**	(0) Não	(1) Sim	(9) Não sabe
**Infarto do coração?**	(0) Não	(1) Sim	(9) Não sabe

EU VOU CITAR UMA LISTA DE EXAMES PARA O RASTREAMENTO DO CÃNCER DE MAMA.

52) O quanto você acredita que cada um desses métodos é efetivo para reduzir a mortalidade por câncer de mama?

Exame clínico de mama

(1) Muito efetivo (2)Pouco efetivo (3) Não é efetivo (4) Efetividade não conhecida (9) Não sei

Auto-exame de mama

(1) Muito efetivo (2)Pouco efetivo (3) Não é efetivo (4) Efetividade não é conhecida (9) Não sei

Mamografia por filme para mulheres entre 40-49 anos

(1) Muito efetivo (2)Pouco efetivo (3) Não é efetivo (4) Efetividade não é conhecida (9) Não sei

Mamografia por filme para mulheres com 50 anos ou mais

(1) Muito efetivo (2)Pouco efetivo (3) Não é efetivo (4) Efetividade não é conhecida (9) Não sei

AGORA EU VOU CITAR UMA LISTA DE EXAMES PARA O RASTREAMENTO DO CÃNCER DE COLO DO ÚTERO.

53) O quanto você acredita que cada um desses métodos é efetivo para reduzir a mortalidade por câncer de colo do útero?

Exame de Papanicolau (convencional ou em base liquida)

(1) Muito efetivo (2)Pouco efetivo (3) Não é efetivo (4) Efetividade não é conhecida (9) Não sei

Inspeção visual com ácido acético ou com solução de lugol forte

(1) Muito efetivo (2)Pouco efetivo (3) Não é efetivo (4) Efetividade não é conhecida (9) Não sei

Teste de HPV

(1) Muito efetivo (2)Pouco efetivo (3) Não é efetivo (4) Efetividade não é conhecida (9) Não sei

AGORA EU VOU CITAR UMA LISTA DE EXAMES PARA O RASTREAMENTO DO CÃNCER DE CÓLON.

54) O quanto você acredita que cada um desses métodos é efetivo para reduzir a mortalidade por câncer de cólon?

Exame de sangue oculto nas fezes

(1) Muito efetivo (2) Pouco efetivo (3) Não é efetivo (4) Efetividade não conhecida (9) Não sei

Sigmoidoscopia

(1) Muito efetivo (2) Pouco efetivo (3) Não é efetivo (4) Efetividade não é conhecida (9) Não sei

Colonoscopia

(1) Muito efetivo (2) Pouco efetivo (3) Não é efetivo (4) Efetividade não é conhecida (9) Não sei

55) Em 2004, o INCA lançou um consenso sobre a frequência recomendada de exames clínicos de mama e mamografias para mulheres sem sintomas, com risco médio ou alto de desenvolver câncer de mama. Na unidade de saúde onde você trabalha você diria que essa diretriz é:

(1) Muito influente (2) Pouco influente (3) Nada influente (9) Não sei

56) O INCA também lançou diretrizes para rastreamento de câncer de colo do útero. Em sua unidade de saúde, você diria que as diretrizes para o rastreamento do câncer de colo do útero são:

(1) Muito influentes (2) Pouco influentes (3) Nada influentes (9) Não sei

57) O INCA também lançou recomendações sobre rastreamento de câncer de cólon. Em sua unidade de saúde, você diria que as diretrizes para o rastreamento do câncer de cólon são:

(1) Muito influentes (2) Pouco influentes (3) Nada influentes (9) Não sei
