# Supplementary material for: Health promoting practices and personal lifestyle behaviors of Brazilian health professionals
Source: BMC Public Health. 2016 Oct 24;16:1114. doi: 10.1186/s12889-016-3778-2 (PMC5078940; doi:10.1186/s12889-016-3778-2)
Supplement: Additional file 1: — Project GUIA’s physicians and nurses survey and community health workers survey. (ZIP 3240 kb) [file 12889_2016_3778_MOESM1_ESM.zip › Project GUIA Survey MDnRNR1.pdf]

**PROJETO GUIA – Inquérito Telefônico nas Unidades Básicas de Saúde**

**Questionário para médicos e enfermeiros**

**BOM** *(DIA, TARDE, NOITE)*. ***MEU NOME É ...., ESTOU LIGANDO DO CENTRO DE EPIDEMIOLOGIA DE PELOTAS-RIO GRANDE DO SUL. ESTAMOS REALIZANDO UMA PESQUISA COORDENADA PELO MINISTÉRIO DA SAÚDE E ALGUMAS UNIVERSIDADES BRASILEIRAS E DOS ESTADOS UNIDOS.***

**Você poderia me informar o primeiro nome de cada um dos** (médicos OU enfermeiros) **que trabalham nessa unidade?**

*COM BASE NOS NOMES INFORMADOS PELO RESPONDENTE, O ENTREVISTADO DEVE "SORTEAR" SEMPRE O NOME DO MEIO. CASO O NÚMERO DE PROFISSIONAIS SEJA PAR, SORTEAR O NOME DO MEIO + 1.*

***GOSTARIA DE FALAR COM O(A)*** *(NOME DO PROFISSIONAL)* ______________________.

Código da UBS: __ __ __ __

City
Cidade: ______________________

State
Estado: __ __

telephone
Telefone: (____) ______________ physician nurse

Profissional entrevistado: (1) Médico(a) (2) Enfermeiro(a)
physician nurse

Data da entrevista: __/__/____

Entrevistador: ______________________ (código do entrevistador: __ __)

**BLOCO DE FORMAÇÃO INICIAL E ATUAÇÃO PROFISSIONAL**

**1) Você estudou até:** You studied until:

(0) Ensino Fundamental (1º grau) incompleto – *PULE PARA QUESTÃO 4* elementary school incomplete or did not finished

(1) Ensino Fundamental (1º grau) completo – *PULE PARA QUESTÃO 4* completed elementary school

(2) Ensino Médio (2º grau) incompleto – *PULE PARA QUESTÃO 4* secondary did not finish

(3) Ensino Médio (2º grau) completo – *PULE PARA QUESTÃO 4* secondary completed

(4) Superior incompleto (undergraduate) advance/university/college incomplete/did not finish

(5) Superior completo (undergraduate) advance/university/college (undergraduate) complete

(6) Pós-graduação post graduation

If you are attending the 3rd degree or already completed, which course did you take?

**2) Se você ESTÁ CURSANDO o 3º GRAU ou já COMPLETOU, qual o curso de graduação?**

(0) Medicina (Medicine) (1) Enfermagem (Nursing) (2) Serviço Social (Social service) (3) Nutrição (nutrition)

(4) Odontologia (dentistry) (5) Fisioterapia (Physiotherapy) (8) NSA ( ) Outro (other) __________

**Há quanto tempo você concluiu o 3º grau: __ __ (anos)** (88) NSA

**3) Você tem:** You have

| | No | Yes | |
|---|---|---|---|
| **Residência médica?** medical residency | (0) Não | (1) Sim | (8) NSA |
| **Residência em enfermagem?** residency in nursing | (0) Não | (1) Sim | (8) NSA |
| **Especialização?** Specialize | (0) Não | (1) Sim | (8) NSA |

SE SIM, QUAL ÁREA: ______________________
if yes, in which area

| | | | |
|---|---|---|---|
| **Mestrado?** masters | (0) Não | (1) Sim | (8) NSA |

SE SIM, QUAL ÁREA: ______________________

| | | | |
|---|---|---|---|
| **Doutorado ?** doctoral degree | (0) Não | (1) Sim | (8) NSA |

[illegible]

4) Qual ano você concluiu a formação de mais alto grau? _ _ _ _

5) Há quanto tempo você trabalha nesta unidade de saúde? _ _ anos _ _ meses
How long have you worked at the health center?

**BLOCO DE VARIÁVEIS DEMOGRÁFICAS**

6) **Qual a sua idade?** _ _ anos what is your age?

7) **Como você se classifica com relação à sua cor de pele?** race/ethnicity/ color of skin?
(1) Branco White (2) Preto Black (3) Amarelo Asian (4) Pardo multiracial (5) Indígena Indigenous (6) Sem declaração undeclared

8) Sexo do entrevistado (1) Masculino (2) Feminino Sex/gender?

9) **Qual é a sua situação conjugal atual?** Marital Status?
(1) Solteiro Single (2) Casado/Mora com companheiro married/with a partner (3) Separado seperated (4) Viúvo widowed or widow[er]

**VARIÁVEIS RELACIONADAS AO ESTADO DE SAÚDE**

What is your height?
10) **Qual a sua altura atual (cm)?** _ _ _ (999) IGN

What is your weight?
11) **Qual o seu peso atual (kg)?** _ _ _ (999) IGN
SOMENTE PARA MULHERES: **Caso a Sra. esteja grávida, indique seu peso antes de engravidar** _ _ _ kg

12) **Em geral, como você considera sua saúde?** In general, you consider your health to be...
**(1) Excelente** excellent **(2) Muito boa** very good **(3) Boa** good **(4) Regular** regular **(5) Ruim** poor/bad

13) **Quantas horas por semana você trabalha nessa unidade de saúde?** _ _ horas por semana

14) **Em uma semana habitual, quantos pacientes você costuma atender?** _ _ _ pacientes (999) Não sabe

15) **Nesta unidade de saúde você atende:**
- **(a) Crianças menores de 2 anos?** (0) Não (1) Sim
- **(b) Crianças entre 2 e 11 anos?** (0) Não (1) Sim
- **(c) Adolescentes entre 12 e 17 anos?** (0) Não (1) Sim
- **(d) Adultos entre 18 e 35 anos?** (0) Não (1) Sim
- **(e) Adultos entre 36 e 59 anos** (0) Não (1) Sim
- **(f) Idosos com 60 anos ou mais** (0) Não (1) Sim

How prepared do you consider yourself to be in regards to talking with patients about the following subjects?
16) **O quanto você se considera preparado para falar com pacientes sobre os seguintes assuntos?Para cada assunto, peço que você responda se você se sente sem preparo, com um pouco de preparo ou bastante preparado.**
- **(a) Nutrição/alimentação** Nutrition/Food (1) Sem preparo unprepared (2) Um pouco de preparo somewhat prepared (3) Bastante preparado very prepared
- **(b) Exercício/atividade física** Exercise/physical activity (1) Sem preparo (2) Um pouco de preparo (3) Bastante preparado
- **(c) Controle de peso** weight control (1) Sem preparo (2) Um pouco de preparo (3) Bastante preparado
- **(d) Câncer de mama** breast cancer (1) Sem preparo (2) Um pouco de preparo (3) Bastante preparado
- **(e) Câncer de colo do útero** cervical cancer of the uterus (1) Sem preparo (2) Um pouco de preparo (3) Bastante preparado

17) **Você costuma avaliar a atividade física de seus pacientes?** (0) Não – *PULE PARA A QUESTÃO 19* (1) Sim

18) **Como você avalia a atividade física de um paciente**

**(a) Faz perguntas gerais sobre a quantidade de atividade física?**
(0) Não (1) Sim (8) NSA

**(b) Faz perguntas gerais sobe a quantidade de atividades sedentárias, como tempo sentado/deitado, assistindo televisão, no computador?**

(0) Não (1) Sim (8) NSA

**(c) Faz perguntas específicas sobre duração, tipo e intensidade das atividades realizadas?**

(0) Não (1) Sim (8) NSA

**(d) Utiliza um questionário padronizado?**

(0) Não (1) SE SIM: **Qual?**__________________ (8) NSA

**(e) Utiliza outro método?**

(0) Não (1) SE SIM: **Qual?**__________________ (8) NSA

---

**19) Quando você atende um paciente com ______________, o que você aconselha?**

when you see a patient with __________, what do you advise?

**a) IMC elevado** (imc) high BMI

| | | |
|---|---|---|
| **Fazer dieta** diet (imc1) | (0) Não | (1) Sim |
| **Praticar atividade física** participate in physical activity (imc2) | (0) Não | (1) Sim |
| **Não fumar** NO/stop smoking (imc3) | (0) Não | (1) Sim |
| **Controlar o uso de álcool** control the use of alcohol (imc4) | (0) Não | (1) Sim |
| **Tomar medicamentos** take medicines (imc5) | (0) Não | (1) Sim |
| | NO | yes |

**b) Dislipidemias / perfil lipídico alterado** dyslipidemias lipid profile changed

| | | |
|---|---|---|
| **Fazer dieta** | (0) Não | (1) Sim |
| **Praticar atividade física** | (0) Não | (1) Sim |
| **Não fumar** | (0) Não | (1) Sim |
| **Controlar o uso de álcool** | (0) Não | (1) Sim |
| **Tomar medicamentos** | (0) Não | (1) Sim |

**c) Hipertensão arterial** hypertension

| | | |
|---|---|---|
| **Fazer dieta** | (0) Não | (1) Sim |
| **Praticar atividade física** | (0) Não | (1) Sim |
| **Não fumar** | (0) Não | (1) Sim |
| **Controlar o uso de álcool** | (0) Não | (1) Sim |
| **Tomar medicamentos** | (0) Não | (1) Sim |

**d) Diabetes tipo 2** Type 2 Diabetes

| | | |
|---|---|---|
| **Fazer dieta** | (0) Não | (1) Sim |
| **Praticar atividade física** | (0) Não | (1) Sim |
| **Não fumar** | (0) Não | (1) Sim |
| **Controlar o uso de álcool** | (0) Não | (1) Sim |
| **Tomar medicamentos** | (0) Não | (1) Sim |

**e) Câncer de mama ou de colo do útero** breast cancer or cervical cancer of the uterus

| | | |
|---|---|---|
| **Fazer dieta** | (0) Não | (1) Sim |
| **Praticar atividade física** | (0) Não | (1) Sim |
| **Não fumar** | (0) Não | (1) Sim |
| **Controlar o uso de álcool** | (0) Não | (1) Sim |
| **Tomar medicamentos** | (0) Não | (1) Sim |
| **Outros** | (0) Não | (1) Sim |

| AS PRÓXIMAS QUESTÕES TRATAM SOBRE ACONSELHAMENTO PARA A PRÁTICA DE ATIVIDADE FÍSICA |
|---|

**Gostaria que você me dissesse se concorda totalmente, concorda, é indeciso(a), discorda ou discorda totalmente com a seguinte frase.**

**20) Programas voltados à atividade física para a comunidade devem ser oferecidos pelo Sistema Único de Saúde**

**(1) Discordo Inteiramente (2) Discordo (3) Indeciso (4) Concordo (5)Concordo inteiramente**

**21) Você considera viável o oferecimento de programas de atividade física na sua unidade de saúde?**

(0) Não (1) Sim

**22) Você considera prioritário o oferecimento de programas de atividade física em sua unidade de saúde?**

(0) Não (1) Sim

**23) Em sua opinião, qual o profissional da saúde é o principal responsável pela promoção da atividade física?**

(1) Médico (2) Profissional de Educação Física (3) Nutricionista (4) Fisioterapeuta

(5) Enfermeiro ( ) Outro ____________________________

**24) Quais destas são dificuldades para você aconselhar sobre a prática de atividade física aos pacientes? Para cada pergunta, por favor, responda sim ou não.**

| | | |
|---|---|---|
| **Falta de conhecimento sobre o assunto** | (0) Não | (1) Sim |
| **Falta de tempo durante o atendimento** | (0) Não | (1) Sim |
| **Falta de locais adequados para a prática** | (0) Não | (1) Sim |

**Outro:**____________________________

**25) Indique uma das alternativas abaixo que melhor represente a sua atitude em relação à orientação de atividade física para a saúde das pessoas que você atende:**

**(1) Eu não recomendo atividade física e não tenho intenção de começar a recomendar**

**(2) Eu não recomendo atividade física, mas estou pensando em começar a recomendar**

**(3) Eu recomendo a atividade física algumas vezes, mas não regularmente**

**(4) Eu recomendo a atividade física regularmente, mas iniciei recentemente**

**(5) Eu recomendo a atividade física regularmente há mais de 6 meses**

**(6) Eu recomendava a atividade física no passado, mas agora não**

BLOCO SOBRE HÁBITOS RELACIONADOS À SAUDE

***NESSA SEÇÃO FAREMOS PERGUNTAS RELACIONADAS AOS SEUS HÁBITOS DE SAUDE***

Normally how many hours per night do you sleep?

**26) Normalmente, quantas horas por noite você dorme?** ____ **horas**

during the past month, approx. how many days did you drink alcohol?

**27) Durante o ultimo mês, aproximadamente, quantos dias você ingeriu bebida alcoólica?**

(0) Nenhum ___ dias

**28) Nesses dias, quantas doses você tomou na ocasião? (1 dose equivale a uma lata de cerveja, 1 taça de vinho, 1 coquetel ou 1 copo de licor)**

(0) ___ doses

**29) Nesse último mês, com que freqüência você tomou 5 ou mais doses na mesma ocasião?**

(0) ___ doses

(0) Não (1) Sim

**31) Em relação ao cigarro, atualmente, você?**

**Não fuma (1) Fuma todos os dias (2) Fuma alguns dias (3) É ex-fumante.**

***AGORA NÓS VAMOS FALAR SOBRE ATIVIDADES FÍSICAS/EXERCÍCIOS QUE VOCÊ REALIZA NO SEU TEMPO LIVRE. POR FAVOR, NÃO RESPONDA AQUI SOBRE AS SUAS ATIVIDADES NO TRABALHO.***

***GOSTARIA QUE VOCÊ PENSASSE EM TODAS AS ATIVIDADES FÍSICAS DE INTENSIDADE FORTE QUE VOCE REALIZOU DESDE*** *<DIA DA SEMANA PASSADA>* ***NO SEU TEMPO LIVRE. ATIVIDADES DE INTENSIDADE FORTE SÃO AQUELAS QUE EXIGEM UM ESFORÇO FÍSICO FORTE, FAZEM VOCE RESPIRAR MAIS RÁPIDO QUE O NORMAL E O CORAÇÃO BATER MAIS FORTE QUE O NORMAL. ESSAS ATIVIDADES PODEM INCLUIR: CORRIDA, JOGAR FUTEBOL, PEDALAR RÁPIDO, ENTRE OUTRAS. PENSE APENAS NAS ATIVIDADES QUE TIVERAM DURAÇÃO DE PELO MENOS 10 MINUTOS SEGUIDOS.***

**32) Desde** *<7 dias atrás>*, **quantos dias você realizou atividades físicas de intensidade forte no seu tempo livre?**

(0) Nenhum – *PULE PARA QUESTÃO 34* __ dias da semana

**33) Nos dias que você realizou essas atividades, aproximadamente, quanto tempo elas duraram por dia?**

__ __ __ minutos (888) NSA

***AGORA PENSE NAS ATIVIDADES FÍSICAS DE INTENSIDADE MODERADA QUE VOCÊ REALIZOU NO SEU TEMPO LIVRE. ATIVIDADES FÍSICAS MODERADAS SÃO AQUELAS QUE EXIGEM UM ESFORÇO FÍSICO MODERADO E FAZEM VOCE RESPIRAR UM POUCO MAIS RÁPIDO QUE O NORMAL. ESTAS PODEM INCLUIR: DANÇAR, PEDALAR DEVAGAR, CAMINHAR, ENTRE OUTRAS. PENSE APENAS NAS ATIVIDADES QUE TIVERAM DURAÇÃO DE PELO MENOS 10 MINUTOS SEGUIDOS.***

**34) Desde** *<7 dias atrás>*, **quantos dias você realizou atividades físicas moderadas no seu tempo livre?**

(0) Nenhum – *PULE PARA QUESTÃO 36* __ dias da semana

**35) Nesses dias, aproximadamente, quanto tempo por dia duraram essas atividades?**

__ __ __ minutos (888) NSA

**36) Desde** *< 7 dias atrás>*, **quantos dias você realizou caminhadas no seu tempo livre?**

(0) Nenhum – *PULE PARA QUESTÃO 38* __ dias da semana

**37) Nesses dias, aproximadamente, quanto tempo duraram essas caminhadas no seu tempo livre?**

__ __ __ **minutos** (888) NSA

***AGORA EU GOSTARIA QUE VOCÊ PENSASSE COMO VOCÊ SE DESLOCA DE UM LUGAR AO OUTRO. PODE SER A IDA E VINDA DO TRABALHO OU QUANDO VOCÊ VAI FAZER COMPRAS, VISITAR AMIGOS OU IR À ESCOLA. LEMBRE DE FALAR APENAS SOBRE AS ATIVIDADES QUE DURAM PELO MENOS 10 MINUTOS SEGUIDOS. NÃO CONSIDERE AQUI AS CAMINHADAS QUE VOCÊ FAZ DURANTE A SUA JORNADA DE TRABALHO.***

**38) Desde** *< 7 dias atrás>*, **quantos dias você utilizou a bicicleta para ir de um lugar para outro?**

(0) Nenhum – *PULE PARA QUESTÃO 40* __ dias da semana

**39) Nesses dias, aproximadamente, quanto tempo duraram essas pedaladas?**

__ __ __ minutos (888) NSA

**40) Desde** *< 7 dias atrás>*, **quantos dias você caminhou para ir de um lugar a outro?**

(0) Nenhum – *PULE PARA QUESTÃO 42* __ dias da semana

**41) Nesses dias, aproximadamente, quanto tempo duraram esses deslocamentos?**

__ __ __ minutos (888) NSA

AGORA VAMOS FALAR SOBRE [illegible]

How much time do you spent watching tv on a normal weekday?

**42) Quanto tempo você passa por dia assistindo TV num dia de semana normal?**

hours

__ __ horas __ __ minutos

how many portions of fruit do you consume per day?

**43) Em média, quantas porções de fruta você consome por dia?**

(0) (1) (2) (3) (4) (5) (6+) — [illegible]

how many portions of vegetables/greens do you consume per day?

**44) Em média, quantas porções de verduras ou vegetais você consome por dia?**

(0) (1) (2) (3) (4) (5) (6+)

***PARA AS QUESTÕES SEGUINTES INDIQUE A RESPOSTA QUE VOCÊ JULGAR CORRETA. CASO VOCÊ NÃO ESTIVER SEGURO DA RESPOSTA, INQUIQUE A OPÇÃO "NÃO SEI".***

**45) O consumo de frutas e verduras que se deve recomendar a um adulto, por dia, é:**

**(1) Mínino 1 porção de fruta e/ou verdura**

**(2) Mínino 2 porções de frutas e/ou verduras**

**(3) Mínino 3 porções frutas e/ou verduras**

**(4) Mínino 4 porções frutas e/ou verduras**

**(5) Mínino 5 porções frutas e/ou verduras**

**(6) Mínino 6 porções frutas e/ou verduras**

**(7) Não sei**

**46) O perímetro abdominal (cintura) recomendado para a América Latina para manter um risco baixo de enfermidade cardiovascular e diabetes tipo 2 é?**

**(1) < 120 cm para homens e de < 110 cm para mulheres**

**(2) < 110 cm para homens e de < 100 cm para mulheres**

**(3) < 100 cm para homens e < de 90 cm para mulheres**

**(4) < 90 cm para homens e < 80 cm para mulheres**

**(5) < 85 cm para homens e < 75 cm para mulheres**

**(6) Não sei**

**47) Como se define sobrepeso e obesidade em adultos?**

**(1) Um IMC > 20 (sobrepeso) e > 25 (obesidade)**

**(2) Um IMC > 25 (sobrepeso) e > 35 (obesidade)**

**(3) Um IMC > 18.5 (sobrepeso) e > 25 (obesidade)**

**(4) Um IMC > 25 (sobrepeso) e > 30 (obesidade)**

**(5) Não sei**

## *SOBRE AS RECOMENDAÇÕES ATUAIS DE ATIVIDADE FÍSICA PARA SAUDE*

**48) Como você considera o seu conhecimento sobre as recomendações atuais de atividade física para a saúde?**

**(1) Sei o suficiente** **(2) Gostaria de aprender mais** **(3) Insuficiente**

**49) Quantos dias da semana, no mínimo, as pessoas devem fazer atividades físicas de intensidade moderada para obter benefícios a saúde?**

__ dias da semana (88) Não importa (99) Não sei

**50) Nos dias em que a pessoa faz atividade física moderada, qual o tempo mínimo recomendado para obter benefícios à saúde?**

__ __ __ minutos (88) Não importa o tempo (99) Não sei

51) Para que uma atividade física moderada tenha efeito positivo sobre a saúde ela deve ser feita de que forma?

(1) Deve ser feita em uma única vez durante o dia

(2) Pode ser feita em uma única vez durante o dia ou dividida em 2-3 vezes, de 10 a 15 minutos, durante o dia

(3) Não sei

52) Quantos dias da semana, no mínimo, as pessoas devem fazer atividades físicas de intensidade forte, para obter benefícios a saúde?

__ dias da semana (88) Não importa (99) Não sei

53) Nos dias em que a pessoa faz atividade física forte, qual o tempo mínimo recomendado para obter benefícios à saúde?

__ __ __ minutos (88) Não importa o tempo (99) Não sei

54) Para que uma atividade física vigorosa tenha efeito positivo sobre a saúde ela deve ser feita de que forma?

(1) Deve ser feita em uma única vez durante o dia

(2) Pode ser feita em uma única vez durante o dia ou dividida em 2-3 vezes, de 10 a 15 minutos, durante o dia

(3) Não sei

Gostaria que você me dissesse se concorda totalmente, concorda, é indeciso(a), discorda ou discorda totalmente com a seguinte frase.

55) As pessoas podem combinar atividades moderadas (exemplo: caminhada) com atividades vigorosas (exemplo: corrida) com o objetivo de atingir a quantidade mínima de atividade física para a saúde.

(1) Discordo Inteiramente (2) Discordo (3) Indeciso (4) Concordo (5)Concordo inteiramente

56) Você acha que a falta de atividade física, sedentarismo, pode causar:

| | | | |
|---|---|---|---|
| Diabetes tipo 2? | (0) Não | (1) Sim | (9) Não sabe |
| Pressão alta? | (0) Não | (1) Sim | (9) Não sabe |
| AIDS? | (0) Não | (1) Sim | (9) Não sabe |
| Osteoporose, fraqueza nos ossos? | (0) Não | (1) Sim | (9) Não sabe |
| Câncer de pulmão? | (0) Não | (1) Sim | (9) Não sabe |
| Depressão? | (0) Não | (1) Sim | (9) Não sabe |
| Cirrose? | (0) Não | (1) Sim | (9) Não sabe |
| Infarto do coração? | (0) Não | (1) Sim | (9) Não sabe |

*EU VOU CITAR UMA LISTA DE EXAMES PARA O RASTREAMENTO DO CÂNCER DE MAMA.*

How much do you believe that each of these methods are an effective way to reduce mortality from breast cancer?

57) O quanto você acredita que cada um desses métodos é efetivo para reduzir a mortalidade por câncer de mama?

Exame clínico de mama — clinical breast exam

(1) Muito efetivo (2)Pouco efetivo (3) Não é efetivo (4) Efetividade não conhecida (9) Não sei

very effective — somewhat effective — not effective — effectiveness not know — I don't know

Auto-exame de mama — self breast exam

(1) Muito efetivo (2)Pouco efetivo (3) Não é efetivo (4) Efetividade não é conhecida (9) Não sei

by mammography film for women between 40-49 year olds

Mamografia por filme para mulheres entre 40-49 anos

(1) Muito efetivo (2)Pouco efetivo (3) Não é efetivo (4) Efetividade não é conhecida (9) Não sei

" "

Mamografia por filme para mulheres com 50 anos ou mais — with 50 or more years of age

(1) Muito efetivo (2)Pouco efetivo (3) Não é efetivo (4) Efetividade não é conhecida (9) Não sei

*AGORA EU VOU CITAR UMA LISTA DE EXAMES PARA O RASTREAMENTO DO CÃNCER DE COLO DO ÚTERO.*

**58) O quanto você acredita que cada um desses métodos é efetivo para reduzir a mortalidade por câncer de colo do útero?**

**Exame de Papanicolau (convencional ou em base liquida)**

**(1) Muito efetivo (2)Pouco efetivo (3) Não é efetivo (4) Efetividade não é conhecida (9) Não sei**

**Inspeção visual com ácido acético ou com solução de lugol forte**

**(1) Muito efetivo (2)Pouco efetivo (3) Não é efetivo (4) Efetividade não é conhecida (9) Não sei**

**Teste de HPV**

**(1) Muito efetivo (2)Pouco efetivo (3) Não é efetivo (4) Efetividade não é conhecida (9) Não sei**

*AGORA EU VOU CITAR UMA LISTA DE EXAMES PARA O RASTREAMENTO DO CÃNCER DE CÓLON.*

How much do you believe that each of these methods are an effective way to reduce mortality from colon cancer?

**59) O quanto você acredita que cada um desses métodos é efetivo para reduzir a mortalidade por câncer de cólon?**

**Exame de sangue oculto nas fezes**
examination of fecal occult blood

**(1) Muito efetivo (2) Pouco efetivo (3) Não é efetivo (4) Efetividade não conhecida (9) Não sei**
very effective / [illegible] effective somewhat or (small effectiveness) / it is not effective / effectiveness is not known / I don't know

**Sigmoidoscopia**
Sigmoidoscopy

(1) Muito efetivo **(2) Pouco efetivo (3) Não é efetivo (4) Efetividade não é conhecida (9) Não sei**
" // " // " // " // " //

**Colonoscopia** Colonoscopy

**(1) Muito efetivo (2) Pouco efetivo (3) Não é efetivo (4) Efetividade não é conhecida (9) Não sei**

~~In 2004 INCA released a consensus about frequent~~ reccomended clinical breast exams & mammographies for women without symptoms as a medical or high risk of developing breast cancer. The health center were you work at ~~would say the guide~~lines are:

**60) Em 2004, o INCA lançou um consenso sobre a frequência recomendada de exames clínicos de mama e mamografias para mulheres sem sintomas, com risco médio ou alto de desenvolver câncer de mama. Na unidade de saúde onde você trabalha você diria que essa diretriz é:**

**(1) Muito influente (2) Pouco influente (3) Nada influente (9) Não sei**
Very influencial / small influence / are not influential / I don't know

**61) O INCA também lançou diretrizes para rastreamento de câncer de colo do útero. Em sua unidade de saúde, você diria que as diretrizes para o rastreamento do câncer de colo do útero são:**

**(1) Muito influentes (2) Pouco influentes (3) Nada influentes (9) Não sei**

INCA also released guidelines for colon cancer screening. In your health center (unit), would you say that the guidelines for screening of colon cancer are:

**62) O INCA também lançou recomendações sobre rastreamento de câncer de cólon. Em sua unidade de saúde, você diria que as diretrizes para o rastreamento do câncer de cólon são:**
very influential / small influence / are not influential / I don't know

**(1) Muito influentes (2) Pouco influentes (3) Nada influentes (9) Não sei**

**Agora eu gostaria de perguntar para você questões específicas sobre exames usados para rastreamento do câncer de mama. Por favor, responda cada questão baseado nas suas recomendações para mulheres sem sintomas e com risco normal pra desenvolver câncer de mama. Se você não realiza rastreamento para câncer de mama em mulheres, simplesmente diga "Eu não realizo rastreamento para câncer de mama em mulheres."**

With which age group do you start screening rootinely for breast cancer?

**63) Com qual idade você começa a rotina para rastreamento do câncer de mama?**
less than 40 / 50 or more

**(1) Menos de 40 anos (2) 40-44 (3) 45-49 (4) 50 ou + anos**

**(5) Eu não faço rastreamento para câncer de mama –** *PULE PARA A QUESTÃO 67*
I don't do a screening for breast cancer.

**64) Com que freqüência você solicita que suas pacientes realizem exame clínico das mamas?**

**A cada __ anos Outra resposta: ______________**
Every _ years other [illegible]

**65) Com que freqüência você solicita que suas pacientes realizem mamografia de rastreamento?**

**A cada __ anos** **Outra resposta: ______________________________**

**66) Existem muitas atividades que fazem parte do rastreamento do câncer de mama. Para cada tipo de serviço que eu vou ler agora gostaria que você indicasse se o serviço é realizado por você, por outros profissionais de saúde, por ninguém ou você não sabe.**

**Ensinar as pacientes a realizarem o auto-exame de mamas**

**(1) Eu realizo** **(2) É realizado por mim e outros profissionais**

**(3) Realizado somente por outros profissionais** **(4) Não é realizado** **(9) Não sei**

**Recomendar acompanhamento para pacientes com resultado positivo no exame clínico de mamas**

**(1) Eu realizo** **(2) É realizado por mim e outros profissionais**

**(3) Realizado somente por outros profissionais** **(4) Não é realizado** **(9) Não sei**

**Recomendar acompanhamento para pacientes com resultado positivo no exame de mamografia**

**(1) Eu realizo** **(2) É realizado por mim e outros profissionais**

**(3) Realizado somente por outros profissionais** **(4) Não é realizado** **(9) Não sei**

**Discutir os resultados da mamografia**

**(1) Eu realizo** **(2) É realizado por mim e outros profissionais**

**(3) Realizado somente por outros profissionais** **(4) Não é realizado** **(9) Não sei**

## *AGORA VAMOS FALAR SOBRE CÂNCER DE CÓLO DO ÚTERO*

**Agora eu gostaria de perguntar para você questões específicas sobre exames usados para rastreamento do câncer de colo do útero. Por favor, responda cada questão baseado nas suas recomendações para mulheres sem sintomas e com risco normal pra desenvolver câncer de colo do útero. Se você não realiza rastreamento para câncer de colo de útero em mulheres, simplesmente diga "Eu não realizo rastreamento para câncer de colo de útero em mulheres."**

**67) Com qual idade você começa a rotina para rastreamento de câncer de colo do útero em mulheres sem sintomas e com risco normal para desenvolver a doença?**

**(1) Menos de 25 anos** **(2) 25-29 anos** **(3) 30-34 anos** **(4) 35 ou + anos** **(5) Outro**

**(6) Eu não faço rastreamento para câncer de colo de útero** – *PULE PARA A QUESTÃO 72*

**68) Com que freqüência você solicita que suas pacientes realizem exame de Papanicolau?**

**A cada __ anos**

**A cada __ anos, SE DOIS EXAMES NORMAIS** (9) Não sabe

**69) Com que freqüência as pacientes deveriam realizar exame de inspeção visual com ácido acético ou com solução de lugol forte?**

**A cada __ anos** (9) Não sabe

70) Com que freqüência as pacientes devem realizar exame de HPV?

A cada __ anos (9) Não sabe

71) Existem muitos serviços que fazem parte do rastreamento do câncer de colo do útero. Para cada tipo de serviço que eu vou ler agora gostaria que você indicasse se o serviço é realizado por você, por outros profissionais de saúde, por ninguém ou você não sabe.

Discutir com os pacientes o resultado do exame de Papanicolau

(1) Eu realizo (2) É realizado por mim e outros profissionais

(3) Realizado somente por outros profissionais (4) Não é realizado (9) Não sei

Acompanhamento de mulheres que apresentem resultados alterados no exame de Papanicolau

(1) Eu realizo (2) É realizado por mim e outros profissionais

(3) Realizado somente por outros profissionais (4) Não é realizado (9) Não sei

Recomendar acompanhamento para mulheres que apresentem resultados alterados no exame de Papanicolau

(1) Eu realizo (2) É realizado por mim e outros profissionais

(3) Realizado somente por outros profissionais (4) Não é realizado (9) Não sei

Relembrar as mulheres sobre as consultas de acompanhamento

(1) Eu realizo (2) É realizado por mim e outros profissionais

(3) Realizado somente por outros profissionais (4) Não é realizado (9) Não sei

*AGORA EU GOSTARIA DE LHE FAZER ALGUMAS PERGUNTAS SOBRE A VACINA CONTRA O HPV*

72) Até o momento o Comitê Permanente de Acompanhamento da Vacina do HPV liderado pelo INCA decidiu não incorporar a vacina contra HPV no Programa Nacional de Imunizações. Se a vacina contra HPV estivesse disponível através do Programa Nacional de Imunizações, você a recomendaria para suas pacientes/clientes

(0) Não (1) Sim – *PULE PARA A QUESTÃO 74*

(9) Não sei – *PULE PARA A QUESTÃO 77*

73) Quais das seguintes razões pelas quais você não recomendaria a vacina contra HPV?

| | | |
|---|---|---|
| Preocupação com relação à segurança da vacina | (0) Não | (1) Sim |
| Preocupação com relação a eficácia da vacina | (0) Não | (1) Sim |
| A vacina poderia estimular comportamentos sexuais arriscados precocemente | (0) Não | (1) Sim |
| Preocupação dos pais sobre a segurança da vacina | (0) Não | (1) Sim |
| Preocupação de pais sobre a eficácia da vacina | (0) Não | (1) Sim |
| Os pais podem achar que a vacina poderia estimular comportamentos sexuais arriscados | (0) Não | (1) Sim |
| Dificuldade em garantir que os pacientes irão completar as três doses da vacina | (0) Não | (1) Sim |

Outra razão: ____________________

**INDIVÍDUOS QUE RESPONDERAM A QUESTÃO 73 DEVEM PULAR PARA A QUESTÃO 77**

**74) Para qual grupo de idade você indicaria a vacina em pessoas do sexo feminino?**

**(1) 9-10 anos** **(2) 11-12 anos** **(3) 13-18 anos** **(4) 19-25 anos** **(5) Maiores de 26 anos**

**75) Você recomendaria a vacina para pessoas do sexo masculino?**

**(0) Não -** *PULE PARA A QUESTÃO 77* **(1) Sim**

**76) Para qual grupo de idade você a indicaria a vacina em pessoas do sexo masculino?**

**(1) 9-10 anos** **(2) 11-12 anos** **(3) 13-18 anos** **(4) 19-25 anos** **(5) Maiores de 26 anos**

***AGORA VAMOS FALAR DE CÂNCER DE CÓLON***

**77) Agora eu gostaria de perguntar sobre o rastreamento do câncer de cólon. Para cada tipo de teste citado eu gostaria que você me indicasse o quanto você está "muito familiarizado", "familiarizado" "pouco familiarizado" ou "não familiarizado". O quanto familiarizado você está com o:**
How familiar are you with:

**Exame de sangue oculto nas fezes (FOBT)**
~~blood test of~~ fecal occult blood test

**(1) Muito familiarizado** **(2) Familiarizado** **(3) Pouco familiarizado** **(4) Não familiarizado**
very familiarize familiarize somewhat not familiarize

**Retosigmoscopia flexível** flexiable sigmoidoscopy familiarize

**(1) Muito familiarizado** **(2) Familiarizado** **(3) Pouco familiarizado** **(4) Não familiarizado**
" " " " " " " "

With which age group do you start a routine screening of colon cancer?
**78) Com qual idade você começa a rotina para rastreamento de câncer de cólon?**

**(1) Menos de 50 anos** **(2) 50 -55anos** **(3) 56-61 anos** **(4) 62-67 anos** **(5) Outro**
less than 50 year olds year olds " " yearolds other

**(6) Eu não faço rastreamento para câncer de cólon** – ENCERRE A ENTREVISTA
I don't do a screening for colon cancer.

**79) Quais dos seguintes exames de rastreamento de câncer de cólon são utilizados na sua unidade para rastrear os pacientes?** Which of the following screening tests for colon cancer are used in your unit (health center) to screen/track patients?

| | No | Yes | |
|---|---|---|---|
| **Exame de sangue oculto nas fezes** | **(0) Não** | **(1) Sim** | (8) NSA not applicable (?) |
| **Sigmoidoscopia** | **(0) Não** | **(1) Sim** | (8) NSA |
| **Colonoscopia** | **(0) Não** | **(1) Sim** | (8) NSA |
