# Supplementary material for: Health promoting practices and personal lifestyle behaviors of Brazilian health professionals
Source: BMC Public Health. 2016 Oct 24;16:1114. doi: 10.1186/s12889-016-3778-2 (PMC5078940; doi:10.1186/s12889-016-3778-2)
Supplement: Additional file 2: — International physical activity questionnaire. (PDF 29 kb) [file 12889_2016_3778_MOESM2_ESM.pdf]

INTERNATIONAL PHYSICAL ACTIVITY QUESTIONNAIRE (October 2002)

LONG LAST 7 DAYS SELF-ADMINISTERED FORMAT

FOR USE WITH YOUNG AND MIDDLE-AGED ADULTS (15-69 years)

The International Physical Activity Questionnaires (IPAQ) comprises a set of 4 questionnaires. Long (5 activity domains asked independently) and short (4 generic items) versions for use by either telephone or self-administered methods are available. The purpose of the questionnaires is to provide common instruments that can be used to obtain internationally comparable data on health–related physical activity.

Background on IPAQ
The development of an international measure for physical activity commenced in Geneva in 1998 and was followed by extensive reliability and validity testing undertaken across 12 countries (14 sites) during 2000. The final results suggest that these measures have acceptable measurement properties for use in many settings and in different languages, and are suitable for national population-based prevalence studies of participation in physical activity.

Using IPAQ
Use of the IPAQ instruments for monitoring and research purposes is encouraged. It is recommended that no changes be made to the order or wording of the questions as this will affect the psychometric properties of the instruments.

Translation from English and Cultural Adaptation
Translation from English is encouraged to facilitate worldwide use of IPAQ. Information on the availability of IPAQ in different languages can be obtained at www.ipaq.ki.se. If a new translation is undertaken we highly recommend using the prescribed back translation methods available on the IPAQ website. If possible please consider making your translated version of IPAQ available to others by contributing it to the IPAQ website. Further details on translation and cultural adaptation can be downloaded from the website.

Further Developments of IPAQ
International collaboration on IPAQ is on-going and an ***International Physical Activity Prevalence Study*** is in progress. For further information see the IPAQ website.

More Information
More detailed information on the IPAQ process and the research methods used in the development of IPAQ instruments is available at www.ipaq.ki.se and Booth, M.L. (2000). *Assessment of Physical Activity: An International Perspective.* Research Quarterly for Exercise and Sport, 71 (2): s114-20. Other scientific publications and presentations on the use of IPAQ are summarized on the website.

INTERNATIONAL PHYSICAL ACTIVITY QUESTIONNAIRE

We are interested in finding out about the kinds of physical activities that people do as part of their everyday lives. The questions will ask you about the time you spent being physically active in the **last 7 days**. Please answer each question even if you do not consider yourself to be an active person. Please think about the activities you do at work, as part of your house and yard work, to get from place to place, and in your spare time for recreation, exercise or sport.

Think about all the **vigorous** and **moderate** activities that you did in the **last 7 days**. **Vigorous** physical activities refer to activities that take hard physical effort and make you breathe much harder than normal. **Moderate** activities refer to activities that take moderate physical effort and make you breathe somewhat harder than normal.

PART 1: JOB-RELATED PHYSICAL ACTIVITY

The first section is about your work. This includes paid jobs, farming, volunteer work, course work, and any other unpaid work that you did outside your home. Do not include unpaid work you might do around your home, like housework, yard work, general maintenance, and caring for your family. These are asked in Part 3.

1. Do you currently have a job or do any unpaid work outside your home?

☐ Yes

☐ No → ***Skip to PART 2: TRANSPORTATION***

The next questions are about all the physical activity you did in the **last 7 days** as part of your paid or unpaid work. This does not include traveling to and from work.

2. During the **last 7 days**, on how many days did you do **vigorous** physical activities like heavy lifting, digging, heavy construction, or climbing up stairs **as part of your work**? Think about only those physical activities that you did for at least 10 minutes at a time.

_____ **days per week**

☐ No vigorous job-related physical activity → ***Skip to question 4***

3. How much time did you usually spend on one of those days doing **vigorous** physical activities as part of your work?

_____ **hours per day**
_____ **minutes per day**

4. Again, think about only those physical activities that you did for at least 10 minutes at a time. During the **last 7 days**, on how many days did you do **moderate** physical activities like carrying light loads **as part of your work**? Please do not include walking.

_____ **days per week**

☐ No moderate job-related physical activity → ***Skip to question 6***

5. How much time did you usually spend on one of those days doing **moderate** physical activities as part of your work?

_____ **hours per day**
_____ **minutes per day**

6. During the **last 7 days**, on how many days did you **walk** for at least 10 minutes at a time **as part of your work**? Please do not count any walking you did to travel to or from work.

_____ **days per week**

☐ No job-related walking → ***Skip to PART 2: TRANSPORTATION***

7. How much time did you usually spend on one of those days **walking** as part of your work?

_____ **hours per day**
_____ **minutes per day**

PART 2: TRANSPORTATION PHYSICAL ACTIVITY

These questions are about how you traveled from place to place, including to places like work, stores, movies, and so on.

8. During the **last 7 days**, on how many days did you **travel in a motor vehicle** like a train, bus, car, or tram?

_____ **days per week**

☐ No traveling in a motor vehicle → ***Skip to question 10***

9. How much time did you usually spend on one of those days **traveling** in a train, bus, car, tram, or other kind of motor vehicle?

_____ **hours per day**
_____ **minutes per day**

Now think only about the **bicycling** and **walking** you might have done to travel to and from work, to do errands, or to go from place to place.

10. During the **last 7 days**, on how many days did you **bicycle** for at least 10 minutes at a time to go **from place to place**?

_____ **days per week**

☐ No bicycling from place to place → ***Skip to question 12***

11. How much time did you usually spend on one of those days to **bicycle** from place to place?

_____ **hours per day**
_____ **minutes per day**

12. During the **last 7 days**, on how many days did you **walk** for at least 10 minutes at a time to go **from place to place**?

_____ **days per week**

☐ No walking from place to place → ***Skip to PART 3: HOUSEWORK, HOUSE MAINTENANCE, AND CARING FOR FAMILY***

13. How much time did you usually spend on one of those days **walking** from place to place?

_____ **hours per day**
_____ **minutes per day**

PART 3: HOUSEWORK, HOUSE MAINTENANCE, AND CARING FOR FAMILY

This section is about some of the physical activities you might have done in the **last 7 days** in and around your home, like housework, gardening, yard work, general maintenance work, and caring for your family.

14. Think about only those physical activities that you did for at least 10 minutes at a time. During the **last 7 days**, on how many days did you do **vigorous** physical activities like heavy lifting, chopping wood, shoveling snow, or digging **in the garden or yard**?

_____ **days per week**

☐ No vigorous activity in garden or yard → ***Skip to question 16***

15. How much time did you usually spend on one of those days doing **vigorous** physical activities in the garden or yard?

_____ **hours per day**
_____ **minutes per day**

16. Again, think about only those physical activities that you did for at least 10 minutes at a time. During the **last 7 days**, on how many days did you do **moderate** activities like carrying light loads, sweeping, washing windows, and raking **in the garden or yard**?

_____ **days per week**

☐ No moderate activity in garden or yard → ***Skip to question 18***

17. How much time did you usually spend on one of those days doing **moderate** physical activities in the garden or yard?

_____ **hours per day**
_____ **minutes per day**

18. Once again, think about only those physical activities that you did for at least 10 minutes at a time. During the **last 7 days**, on how many days did you do **moderate** activities like carrying light loads, washing windows, scrubbing floors and sweeping **inside your home**?

_____ **days per week**

☐ No moderate activity inside home → ***Skip to PART 4: RECREATION, SPORT AND LEISURE-TIME PHYSICAL ACTIVITY***

19. How much time did you usually spend on one of those days doing **moderate** physical activities inside your home?

_____ **hours per day**
_____ **minutes per day**

PART 4: RECREATION, SPORT, AND LEISURE-TIME PHYSICAL ACTIVITY

This section is about all the physical activities that you did in the **last 7 days** solely for recreation, sport, exercise or leisure. Please do not include any activities you have already mentioned.

20. Not counting any walking you have already mentioned, during the **last 7 days**, on how many days did you **walk** for at least 10 minutes at a time **in your leisure time**?

_____ **days per week**

☐ No walking in leisure time → ***Skip to question 22***

21. How much time did you usually spend on one of those days **walking** in your leisure time?

_____ **hours per day**
_____ **minutes per day**

22. Think about only those physical activities that you did for at least 10 minutes at a time. During the **last 7 days**, on how many days did you do **vigorous** physical activities like aerobics, running, fast bicycling, or fast swimming **in your leisure time**?

_____ **days per week**

☐ No vigorous activity in leisure time → ***Skip to question 24***

23. How much time did you usually spend on one of those days doing **vigorous** physical activities in your leisure time?

_____ **hours per day**
_____ **minutes per day**

24. Again, think about only those physical activities that you did for at least 10 minutes at a time. During the **last 7 days**, on how many days did you do **moderate** physical activities like bicycling at a regular pace, swimming at a regular pace, and doubles tennis **in your leisure time**?

_____ **days per week**

☐ No moderate activity in leisure time → ***Skip to PART 5: TIME SPENT SITTING***

25. How much time did you usually spend on one of those days doing **moderate** physical activities in your leisure time?
_____ **hours per day**
_____ **minutes per day**

PART 5: TIME SPENT SITTING

The last questions are about the time you spend sitting while at work, at home, while doing course work and during leisure time. This may include time spent sitting at a desk, visiting friends, reading or sitting or lying down to watch television. Do not include any time spent sitting in a motor vehicle that you have already told me about.

26. During the **last 7 days**, how much time did you usually spend **sitting** on a **weekday**?

_____ **hours per day**
_____ **minutes per day**

27. During the **last 7 days**, how much time did you usually spend **sitting** on a **weekend day**?

_____ **hours per day**
_____ **minutes per day**

This is the end of the questionnaire, thank you for participating.
